# Regulatory Roles of Six-Transmembrane Epithelial Antigen of the Prostate Family Members in the Occurrence and Development of Malignant Tumors

**DOI:** 10.3389/fcell.2021.752426

**Published:** 2021-10-29

**Authors:** Wen-Jia Chen, Hua-Tao Wu, Chun-Lan Li, Yi-Ke Lin, Ze-Xuan Fang, Wen-Ting Lin, Jing Liu

**Affiliations:** ^1^Changjiang Scholar’s Laboratory/Guangdong Provincial Key Laboratory for Diagnosis and Treatment of Breast Cancer/Department of Physiology, Shantou University Medical College, Shantou, China; ^2^Department of General Surgery, The First Affiliated Hospital of Shantou University Medical College, Shantou, China; ^3^Department of Pathology, Shantou University Medical College, Shantou, China

**Keywords:** STEAP, cancer, reductase, immunotherapy, biomarkers

## Abstract

The human six-transmembrane epithelial antigen of the prostate (STEAP) proteins, which include STEAP1–4 and atypical STEAP1B, contain six transmembrane domains and are located in the cell membrane. STEAPs are considered archaeal metal oxidoreductases, based on their heme groups and F420H2:NADP+ oxidoreductase (FNO)-like structures, and play an important role in cell metal metabolism. Interestingly, STEAPs not only participate in biological processes, such as molecular transport, cell cycling, immune response, and intracellular and extracellular activities, but also are closely related to the occurrence and development of several diseases, especially malignant tumors. Up to now, the expression patterns of STEAPs have been found to be diverse in different types of tumors, with controversial participation in different aspects of malignancy, such as cell proliferation, migration, invasion, apoptosis, and therapeutic resistance. It is clinically important to explore the potential roles of STEAPs as new immunotherapeutic targets for the treatment of different malignant tumors. Therefore, this review focuses on the molecular mechanism and function of STEAPs in the occurrence and development of different cancers in order to understand the role of STEAPs in cancer and provide a new theoretical basis for the treatment of diverse cancers.

## Introduction

The six-transmembrane epithelial antigen of the prostate (STEAP) family proteins belong to a class of cellular transmembrane proteins. With five members so far, namely STEAP1–4 and STEAP1B, STEAP proteins possess a similar structure comprised of 4–6 transmembrane domains and intracellular amino- and carboxyl-terminal domains (Hubert et al., 1999; Rinaldy and Steiner, 1999; Zhang et al., 2001; Korkmaz et al., 2002, 2005; Grunewald et al., 2012a; Gomes et al., 2014b). Except for the highly homologous STEAP1 and STEAP1B, all other members have general metal reductase activity catalyzed by an F420H2:NADP+ oxidoreductase (FNO)-like domain (Knutson, 2007). However, it has been reported that the intramembrane heme group in STEAP1 and STEAP1B can still be involved in metal metabolism, especially for the reduction and absorption of iron and copper (Ohgami et al., 2006).

Not surprisingly, STEAP family proteins have been reported to affect both intracellular oxidative stress and inflammation, by reducing extracellular Fe^3+^/Cu^2+^ to Fe^2+^/Cu^+^, and cell uptake of Fe^2+^/Cu^+^ through transferrin and copper transport proteins, as well as other cellular biological processes (Ohgami et al., 2005; Liao et al., 2020; Wu et al., 2020). In addition, the abnormal expression of STEAP family members in malignancies has been reported to be related to cell proliferation, migration, invasion, apoptosis, and prognosis by activating or suppressing different signaling pathways. Importantly, the location and function of STEAP family members in cells and their differential expression in tumor tissues raise awareness of the roles of STEAPs in tumorigenesis and development. This review focuses on the current literature, discusses the expression and functions of STEAP family members in different tumors, and describes their roles in tumors in order to propose therapeutic strategies for the treatment of malignant tumors.

## Structural Characteristics of Six-Transmembrane Epithelial Antigen of the Prostate

To better understand the basic characteristics of STEAP family, the information for each gene was obtained from the National Biotechnology Information Center (Table 1). STEAP1, usually referred to as STEAP, is the first reported STEAP protein and is located on chromosome 7q21.13. The STEAP1 gene has a total length of 10,359 bp and is comprised of 5 exons (Hubert et al., 1999). Only one mRNA transcript (NM_012449.3) has been recorded, is 1,219 nt in length, and encodes a 331-amino acid STEAP1 protein (NP_036581.1). The STEAP2 (also known as STAMP1) gene is also located on chromosome 7q21.13 and has a total length of 31,669 bp and 9 exons. The STEAP2 gene can be transcribed into six different mRNAs, the longest (NM_152999.4) of which is 6,871 nt in length and encodes the largest STEAP2 protein (NP_694544.2) at 490 amino acids. Other mRNA transcripts result in shorter encoded proteins. The gene for STEAP3, also known as pHyde, TSAP6, or STAMP3, is located on chromosome 2q14.2 and has a total length of 43,177 bp and 11 exons. The STEAP3 gene produces four mRNA transcripts of varying lengths, with the longest (NM_182915.3) being 4,259 nt and encoding the largest STEAP3 protein (NP_878919.2) at 498 amino acids. Other mRNA transcripts lack certain exons, resulting in shorter encoded proteins. Because the sequence of STEAP4 is similar to that of STAMP1 (STEAP2), it is also named STAMP2 and was identified in 2005 (Korkmaz et al., 2005). The STEAP4 gene is located on chromosome 7q21.12 and encodes a total length of 36,003 bp and 6 exons. Three different mRNA transcripts are produced by the STEAP4 gene. Among them, the main mRNA transcript (NM_024636.4) is 9,991 nt long, while the longest mRNA transcript (NM_001205315.2) contains additional exons compared with the principal transcript, but encodes the same 459-amino acid protein (NP_078912.2). Other mRNA transcripts lack specific coding exons, resulting in shorter encoded proteins. STEAP1B is a newly discovered member of the STEAP family and has 88% homology with STEAP1 (Grunewald et al., 2012a), but its structure is different from that of other family members. The gene for STEAP1B is located on chromosome 7p15.3 and has a total length of 80,839 bp and 6 exons. Transcription of the STEAP1B gene produces two mRNA transcripts. The longer one (NM_001164460.1), namely STEAP1B1, is comprised of 1,299 nt and encodes a protein of 342 amino acids (NP_001157932.1), while the shorter one (STEAP1B2, NM_207342.2) uses an alternate in-frame splice site in the 5′ coding region and an alternate 3′ exon with a distinct 3′ coding region and 3′UTR compared to the longer one. The resulting protein lacks an internal segment near the N-terminus and has a shorter and distinct C-terminus.

**TABLE 1:** Members of the STEAP family.

**Gene**	**Alternative names**	**Location**	**Size (bp)**	**Exons**	Transcript^*^ (nt)	Protein^*^ (aa)
STEAP1	PRSS24 STEAP	7q21.13	10,359	5	NM_012449.3 (1,219)	NP_036581.1 (339)
STEAP2	STMP IPCA1 PUMPCn STAMP1 PCANAP1	7q21.13	31,669	9	NM_001244944.2 (6,948)	NP_001035755.1 (490)
					NM_152999.4 (6,871)	NP_001035756.1 (454)
					NM_001040665.2 (6,821)	NM_001244946.2 (451)
					NM_001040666.1 (2,230)	
					NM_001244945.2 (2,645)	
					NM_001244946.2 (2,267)	
STEAP3	STMP3 TSAP6 pHyde AHMIO2 Dudlin-2 Dudulin-2	2q14.2	43,177	11	NM_182915.3 (4,259)	NP_878919.1 (498)
					NM_018234.3 (3,912)	NP_001008410.1 (488)
					NM_001008410.2 (3,844)	NP_619543.1 (457)
					NM_138637.3 (4,076)	
STEAP4	TIARP STAMP2 SchLAH TNFAIP9	7q21.12	36,003	6	NM_024636.4 (9,991)	NP_001192244.1 (459)
					NM_024636.4 (10,090)	NP_001192245.1 (283)
					NM_024636.4 (9,463)	
STEAP1B	–	7p15.3	80,839	6	NM_001164460.1 (1,299)	NP_001157932.1 (342)
					NM_207342.2 (1,298)	NP_997225.1 (245)

*Gene information obtained from the National Center for Biotechnology Information.

The structures of STEAP family proteins are similar. From the perspective of cell localization, all STEAP family proteins are located on the cell membrane and in the cytoplasm. The STEAP1–4 proteins located on the surface of the plasma membrane all have six potential transmembrane regions, as well as an intracellular hydrophilic amino and carboxyl-terminal, indicating that the STEAP proteins may function as a channel or transporter (Hubert et al., 1999). Furthermore, STEAP2 and STEAP4 are also located in vesicle-tubular structures of the trans-Golgi body network, plasma membrane, and cytoplasm, shuttle between the plasma membrane and Golgi body, and co-localize with early endosomal antigen 1 (EEA1), thereby participating in the secretion/endocytosis pathway (Korkmaz et al., 2005). STEAP3 is mainly located on the plasma and intracellular membranes (Valadi et al., 2007; Lespagnol et al., 2008). The protein structure of STEAP1 and STEAP2 contains at least one heme group, in the membrane, that may be related to the absorption of iron and copper (Finegold et al., 1996; Gomes et al., 2012). STEAP2, STEAP3, and STEAP4 have intrinsic metal reductase activity, endowed by its paleontological and bacterial FNO-like domain (Finegold et al., 1996), for transfer of electrons, which is necessary for iron and copper uptake (Passer et al., 2003; Ohgami et al., 2005). In addition, STEAP4 also has six-transmembrane domains, with the COOH end and the NH end containing dinucleotide binding regions, an NADP REDOX motif, and a pyrrolidine 5-carboxylate reductase motif, which may also be involved in the metal redox process (Korkmaz et al., 2005). However, different from other members, STEAP1 cannot reduce metals as it lacks the NADP+ oxidoreductase FNO-like domain homologous to paleontological and bacterial F420H2 (Ohgami et al., 2005). Nevertheless, it has been suggested that STEAP1 may be involved in iron metabolism, as it coexists with other iron uptake endosomal proteins, such as transferrin (Ohgami et al., 2006). The structure of STEAP1B is different from other proteins. STEAP1B has only four potential transmembrane regions and intracellular COOH and NH2 terminal regions (Gomes et al., 2014b). Without an NADPH oxidoreductase domain and a heme-binding site, STEAP1B is predicted to lack oxidoreductase activity (Grunewald et al., 2012a).

## Diverse Expression Patterns of Six-Transmembrane Epithelial Antigen of the Prostate and Their Regulatory Mechanisms in Cancers

It is commonly found that STEAP1 is up-regulated in a variety of tumor tissues, especially in prostate cancer. Currently, investigations of STEAP1 mainly focus on prostate cancers and show that expression of STEAP1 is elevated compared with normal prostate tissue (Li et al., 2004; Challita-Eid et al., 2007; Wong and Abubakar, 2010; Hayashi et al., 2011; Gomes et al., 2013, 2014a, 2018; Ihlaseh-Catalano et al., 2013; Whiteland et al., 2014). The mutual regulation has been reported between STEAP1 and sex hormones. Gomes et al. (2013) showed that 5α-dihydrotestosterone (DHT) or 17β-estradiol (E2) treatment of prostate cancer cells suppresses the expression of STEAP1, but that this down-regulation of STEAP1 is AR-dependent and ER-independent. In addition, up-regulated STEAP1 expression has also been observed in renal cell carcinoma, bladder cancer, Ewing’s sarcoma, breast cancer, colorectal cancer (CRC), gastric cancer, ovarian cancer, and lung cancer (Maia et al., 2008; Azumi et al., 2010; Grunewald et al., 2012b; Zhuang et al., 2015; Wu et al., 2018; Nakamura et al., 2019; Guo Q. et al., 2020; Jiang et al., 2020; Jiao et al., 2020; Tian et al., 2020). Phosphorylated eIF4E is required for peritoneal metastasis of gastric cancer *via* initiating the cap-dependent translation of STEAP1, providing phosphorylation of eIF4E as an effective therapeutic target for patients with peritoneal metastasis through translational control of STEAP1 (Jiang et al., 2020). Importantly, high STEAP1 levels are associated with low overall survival (OS) rates of patients with prostate cancer, CRC, lung cancer, ovarian cancer, diffuse large B-cell lymphoma, acute myeloid leukemia and multiple myeloma (Moreaux et al., 2012; Ihlaseh-Catalano et al., 2013; Lee et al., 2016; Guo Q. et al., 2020; Jiao et al., 2020), suggesting the prognostic value of STEAP1 as a biomarker of cancer. At present, the carcinogenic effect of STEAP1 in tumor progression is being studied intensely. However, certain investigations have also shown that STEAP1 also plays a role in inhibiting tumor growth. Xie et al. (2019) compared the expression of STEAP1 in normal breast tissue (*n* = 40), benign fibroadenoma (*n* = 52), and primary breast cancer (*n* = 211), and found a low level of STEAP1 in primary breast cancer tissues. In the MCF-7 breast cancer cell line, treatment with E2 also reduces the expression of STEAP1 but is mediated by membrane-bound ERalpha (mbERalpha) (Maia et al., 2008). It is speculated that the carcinogenic and anti-cancer effects of STEAP1 in diverse types of cancer may be related to different hormone levels and regulation of hormone receptor locally or throughout the body. Furthermore, Sun et al. (2019) also showed down-regulation of STEAP1 expression in endometrial carcinoma cell lines compared with normal endometrial cells, and low expression of STEAP1 has been related to poor prognosis of patients with Ewing’s sarcoma, CRC, and breast cancer (Grunewald et al., 2012c; Lee et al., 2016; Xie et al., 2019). Although reports on down-regulation of STEAP1 expression in tumor tissues are limited, they evoke further studies to investigate a potential anti-cancer role of STEAP1 in cancers.

Studies on STEAP2 expression in cancers have also mainly focused on prostate cancers, although studies are limited. STEAP2 expression in prostate cancer was found to be significantly higher than that in normal tissues (Porkka et al., 2002; von Rozycki et al., 2004; Wang et al., 2010; Whiteland et al., 2014; Burnell et al., 2018, 2019). Specifically, Korkmaz et al. (2002) revealed that STEAP2 is highly expressed in androgen-sensitive, androgen receptor-positive prostate cancer cells, but not in androgen receptor-negative prostate cancer cells, suggesting that the regulation of STEAP2 expression may be related to sex hormone signaling. With regard to the molecular mechanism of STEAP2 in prostate cancers, Gonen-Korkmaz et al. (2014) overexpressed STEAP2, in AR-negative DU145 prostate cancer cells, and showed an NFκB-mediated downregulation of STEAP2 expression following treatment with tumor necrosis factor-α (TNF-α). The authors showed that NFκB silencing increased anti-apoptotic STEAP2 expression, as well as inhibited p53 and MDM2 expression in TNF-α-treated, STEAP2-overexpressing DU145 cells, suggesting inhibition of NFκB for prostate cancer prevention in specific patients (Gonen-Korkmaz et al., 2014). Interestingly, Wang et al. (2010) found that activation of extracellular signal-regulated kinase, implicated in prostate cancer progression, increased ectopic expression of STEAP2 in AR-negative DU145 cells, but decreased STEAP2 levels in AR-positive LNCaP cells, suggesting a potential interaction between STEAP2 and sex hormones. The expression of STEAP2 was also found to be up-regulated in colon cancers (Bhatlekar et al., 2014). Similar to the expression pattern of STEAP1, STEAP2 also has been reported to play conflicting roles in prostate and breast cancers, Yang et al. (2020) proposed STEAP2 to be a tumor suppressor in breast cancers, based on its low expression in breast cancer. Through bioinformatics methods, Liu et al. (2021) also predicted that the expression of STEAP2 in glioblastoma was down-regulated compared with normal brain tissue. To construct a multiple RNA-based prediction model in patients with ovarian cancer, Zhang et al. (2020) used sequencing data from The Cancer Genome Atlas (TCGA) database and identified TM4SF1-AS1-miR-186-STEAP2 and LINC00536-miR-508-STEAP2 as new interaction axes to explain the possible functions of these RNAs in the prediction model for disease-free survival in patients with ovarian cancer. However, further investigation of these new axes is required for verification (Zhang et al., 2020).

STEAP3 was first found in prostate tissues and proposed as a candidate for prostate cancer immunotherapy (Machlenkin et al., 2005). Although the expression of STEAP3 in poorly differentiated prostate cancer is lower than that in well-differentiated and moderately differentiated prostate cancer, there is no difference in STEAP3 expression between benign prostatic hyperplasia and prostate cancer (Porkka et al., 2003). It was also found that the expression of STEAP3 is up-regulated in glioma, bladder cancer, and colon cancer (Isobe et al., 2011; Kim et al., 2016; Weston et al., 2016; Han et al., 2018; Zhang et al., 2019), whereas the expression of STEAP3 in hepatocellular carcinoma (HCC), breast cancer, and lung cancer is lower than that in normal tissues (Boelens et al., 2009; Caillot et al., 2009; Savci-Heijink et al., 2016; Cadiou et al., 2017). Han et al. (2018) reported that the expression of STEAP3 in gliomas is negatively correlated with patient OS, and multivariate Cox regression analysis showed that STEAP3 is an independent prognostic indicator. Zhang et al. (2019) used TCGA and GSE16011 online datasets to show that overexpression of STEAP3 is associated with poor prognosis in patients with glioblastoma. Research focused on the regulation of STEAP3 in cancers is limited. In a transcriptome analysis of a CRC series, an increased level of membrane copper transporter 1 protein (CTR1) accompanied increased STEAP3 transcription (Barresi et al., 2016). Yu et al. (2020) demonstrated that in pancreatic cancer, heat shock protein family B member 2 (HSPB2) could combine with mutant p53 to change the DNA binding site of mutant p53, resulting in an upregulated level of STEAP3 and leading to an inhibition of both cell proliferation and angiogenesis.

As a relatively new member of the STEAP family, STEAP4 was found to be increased in human prostate cancers compared with normal prostate tissues (Korkmaz et al., 2005; Jin et al., 2015), and is up-regulated in human colon cancer cells, predicting poor prognosis of patients with colon cancers (Xue et al., 2017). In CRC, interleukin-17 (IL-17) drives cellular uptake of copper through upregulation of STEAP4 expression, and the proinflammatory cytokines interleukin (IL)-6 and IL-1β can synergistically increase androgen-induced STEAP4 expression in prostate cancer cells, with knockdown of STEAP4 enhancing the ability of IL-6 and IL-1β to inhibit cell growth (Pihlstrom et al., 2021). In addition, IL-17 and TNF-α rely heavily on TNF receptor associated factor 4 (TRAF4) to up-regulate the expression of STEAP4 and thus play a role in airway epithelial cells (Jiang et al., 2021). Interestingly, although the up-regulated expression of STEAP4 was also detected in alcohol-induced breast cancer cells (Gelfand et al., 2017), Wu et al. (2020) found that the mRNA level of STEAP4 was decreased in tissues of ductal breast carcinoma compared with normal tissues. In bladder cancers, low STEAP4 expression was found in cancer tissues compared with normal, and the circular RNA circPICALM competed with STEAP4 for binding to miR-1265 to eliminate the enhancing effect of miR-1265 on invasion (Yan et al., 2019). Meanwhile, bioinformatics analysis has also revealed that the transcription level of STEAP4 in head and neck cancer tissues is reduced compared with normal tissues, and a low level of STEAP4 resulted in poor disease-free survival, progression-free survival and OS (Lan et al., 2021).

Currently, the research on STEAP1B in tumor is very limited. STEAP1B1 and STEAP1B2 mRNA are differentially expressed in prostate cell lines. Non-neoplastic prostate cells show little to no STEAP1B1 and STEAP1B2 mRNA expression. On the other hand, in malignant prostate cells, LNCaP and PC3, STEAP1B2 is highly expressed, whereas STEAP1B1 mRNA is mainly expressed on PNT2 and PC3 cells, and under-expressed on LNCaP cells (Gomes et al., 2014b).

## Biological Functions and Related Molecular Mechanisms of Six-Transmembrane Epithelial Antigen of the Prostate in Cancers

**FIGURE 1:**
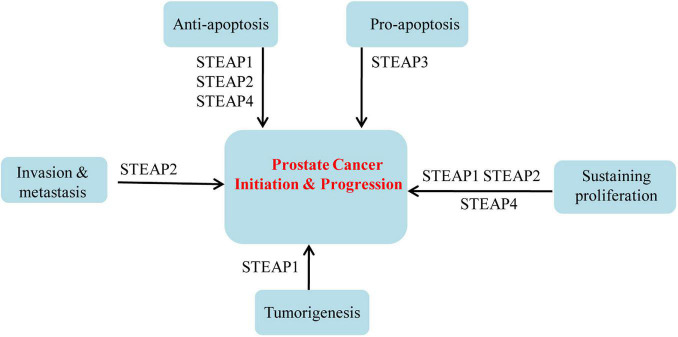
Roles of STEAPs in prostate cancers.

**FIGURE 2:**
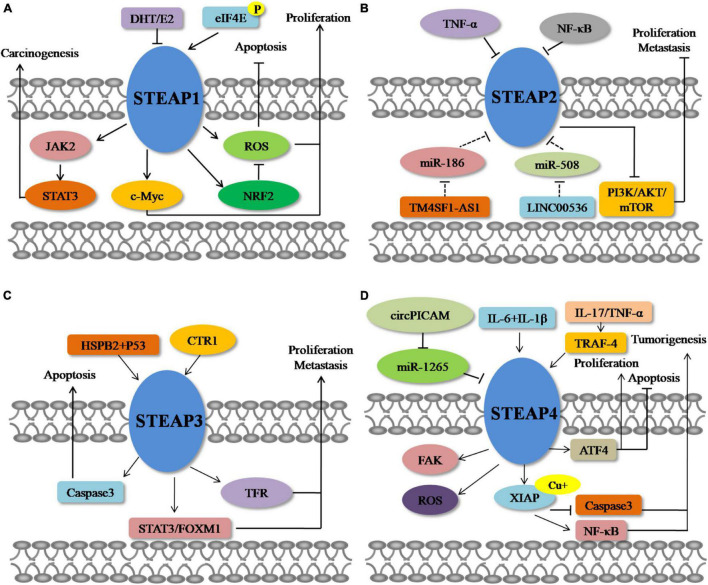
Molecular mechanisms of STEAPs in cancer. **(A)** STEAP1. **(B)** STEAP2. **(C)** STEAP3. **(D)** STEAP4.

Based on the above summary of differential expression patterns of STEAP1 in a variety of tumors, dysregulated STEAP1 affects the occurrence and development of different types of cancers (Figure 1). Some investigations evoke an oncogenic role for STEAP1 in cancers. Yamamoto et al. (2013) found that in prostate cancer, STEAP1, as a cell surface membrane protein, is required for intercellular communication between tumor cells and adjacent tumor stromal cells to augment tumor growth. In androgen-dependent prostate cancers, Gomes et al. (2018) showed that knockdown of STEAP1 inhibits cell growth and induces apoptosis of LNCaP prostate cancer cells in a DHT-independent manner. In lung cancers, STEAP1 was found to affect endothelial cell migration and angiogenesis, and knockout of STEAP1 could inhibit the proliferation, migration, and invasion of tumor cells *via* the JAK2/STAT3 signaling pathway (Zhuang et al., 2015; Tian et al., 2020). Based on transcriptome and proteome analyses, Grunewald et al. (2012b) showed that STEAP1 levels correlate with oxidative stress responses and elevated reactive oxygen species (ROS), which regulate redox-sensitive and proinvasive genes to promote tumor proliferation and invasive behavior, and that knockout of STEAP1 reduces ROS levels and inhibits Ewing tumor proliferation, anchorage-independent colony formation, invasion *in vitro* and metastasis *in vivo*. Oxidative stress and ROS levels are also suppressed in CRC cells, by STEAP1 silencing, through regulation of the nuclear erythroid 2-related factor (NRF2) transcription factor, resulting in reduced cell growth and elevated apoptosis (Nakamura et al., 2019). In gastric cancers, STEAP1 was shown to promote peritoneal metastasis, and up-regulation of STEAP1 was found to promote tumor proliferation, migration, invasion, and tumorigenicity (Wu et al., 2018; Jiang et al., 2020). Silencing of STEAP1 suppressed c-Myc expression in hepatocarcinoma to arrest cancer cells in G1 phase and inhibit cell proliferation (Iijima et al., 2021). Jiao et al. (2020) found a relationship between high STEAP1 levels and epithelial-mesenchymal transition (EMT)-related genes, and demonstrated that STEAP1 promotes ovarian cancer metastasis by aiding EMT progression. Interestingly, down-regulation of STEAP1 in this study inhibited the invasion, migration, proliferation, clone formation, and EMT progression of human ovarian cancer cells and the growth of xenografts *in vivo*, but promoted apoptosis at the same time (Jiao et al., 2020). Although an oncogenic role of STEAP1 in tumors is commonly observed, a tumor suppressive function has also been reported. Recently, Xie et al. (2019) showed that knockdown of STEAP1 expression enhances breast cancer cell invasiveness and migration, and is accompanied by increased expression of EMT-related genes, MMP2, MMP9, MMP13, VIM, and CDH2, as well as decreased CDH1 expression. In a tumor of the female reproductive system, STEAP1 was restrictively expressed in endometrial carcinoma, and down-regulation of STEAP1 increased cancer cell proliferation, colony formation, migration, invasion, and EMT progression of endometrial carcinoma (Sun et al., 2019) (Figure 2).

An increased level of STEAP2 in prostate cancer suggests an oncogenic role. Wang et al. (2010) investigated STEAP2 function in prostate cancer and found that knockdown of STEAP2 dramatically retarded proliferation *in vitro* and *in vivo*, and interestingly, increased expression of STEAP2 was induced by the activation of extracellular signal-regulated kinase (ERK1/2). Whiteland et al. (2014) found that overexpression of STEAP2 resulted in the gain of ability to migrate and invade in normal prostate cells, and Burnell et al. (2018) showed that genes associated with STEAP2 enhancement of invasion were MMP3, MMP10, MMP13, FGFR4, IL-1β, KiSS1, and SERPINE1 in PC3 cells, and MMP7 in LNCaP cells, with altered CD82 in both. In contrast, in breast cancer, STEAP2 hinders cellular proliferation, invasion, and metastasis by inhibiting EMT through suppressing the PI3K/AKT/mTOR signaling pathway (Guo H. et al., 2020), predicting a tumor suppressor role in breast cancers.

The function of STEAP3 in tumors is also controversial. Zhang et al. (2001) first reported that STEAP3 induced apoptosis of prostate cells, based on a dose-dependent stimulation of endogenous caspase-3 expression. However, in glioblastoma, loss of STEAP3 attenuated the aggressive phenotype of glioma cells in regard to cell proliferation, invasion, sphere formation *in vitro*, and tumor growth *in vivo*, through suppressing mesenchymal transition, transferrin receptor expression and STAT3–FoxM2 signaling (Han et al., 2018). In biopsies from patients with HCC, decreased expression of STEAP3 was observed in cirrhotic tissues and related to decreased apoptosis, serving as a warning sign and predictive biomarker for the development of HCC (Caillot et al., 2009).

STEAP4, a relatively new STEAP, has been demonstrated to promote prostate cancer, based on its ability to increase cell growth and colony formation (Korkmaz et al., 2005). Jin et al. (2015) reported that STEAP4 increases ROS in prostate cancer cells through its iron reductase activity, and subsequently affects proliferation, colony formation, anchorage-independent growth, and apoptosis, involving a mechanism at least partly mediated by activating transcription factor 4 (ATF4). Recently, Orfanou et al. (2021) investigated the function of STEAP4 in breast cancer and demonstrated that knockdown of STEAP4 suppresses cell proliferation and enhances the inhibition of lapatinib in HER2-overexpressing breast cancer, predicting an oncogenic role for STEAP4 in breast cancer. On the contrary, STEAP4-mediated increases in intracellular copper levels result in activation of the E3 ligase X-linked inhibitor of apoptosis (XIAP), enhancing IL-17-induced NF-κB activation and inhibiting caspase-3 activity (Liao et al., 2020). However, in bladder cancer, the circular RNA circPICALM competed with STEAP4 for binding to miR-1265 and eliminated the ability of miR-1265 to enhance invasion (Yan et al., 2019).

Up to now, there are no relevant reports regarding the function of STEAP1B in cancer. However, considering the similar structural features of STEAP1B with other family members, STEAP1B may play a role in different cancers, but experimental proof is still required.

## Therapeutic Implications of Six-Transmembrane Epithelial Antigen of the Prostate in Cancers

Considering their roles and location in the cell membrane, STEAPs are currently considered as promising therapeutic targets for cancers, especially prostate cancers. Therapeutic strategies have been discovered and developed during the last few years for targeting STEAP1, including monoclonal antibodies (mAbs), antibody-drug conjugates, DNA vaccines, and small non-coding RNAs (ncRNAs) (Barroca-Ferreira et al., 2018).

Challita-Eid et al. (2007) first generated two mAbs that bind to cell surface STEAP1 epitopes, and both of them inhibited STEAP1-induced intercellular communication in a dose-dependent manner to suppress tumor growth *in vivo*. Subsequently, Esmaeili et al. (2018) developed a single-chain fragment variable (scFv) antibody, against a STEAP1 epitope, that successfully inhibited intercellular communication between prostate cancer cells by blocking gap junctions between cells, demonstrating a high potential for mAbs or single-chain antibodies as effective agents for prostate cancer immunotherapy. To combine the specificity of antibodies with cytotoxic potency of chemotherapeutic drugs, Boswell et al. (2011) designed and produced a synthetic antitumor drug involving humanized anti-STEAP1-monomethylauristatin E (MMAE) conjugates to increase hepatic uptake and reduce drug levels in other highly vascularized organs. Based on a high expression level of STEAP1, Yuan et al. (2019) established a novel contrast agent for ultrasound imaging by conjugating biotinylated STEAP1 monoclonal antibodies with streptavidin-coated SonoVue microbubbles, providing a prospective method to identify prostate tumors effectively *in vivo*. These experiments suggest that STEAP1 targeting may be an attractive and selective way to deliver drugs to cancer cells.

In addition, immunotherapy may provide an alternative treatment for cancer patients, especially for tumors with overexpressed antigens that can be recognized by immune cells. Azumi et al. (2010) used STEAP-derived epitope peptides to stimulate T cells, in the context of multiple major histocompatibility complex class II alleles, and induce STEAP-specific helper T lymphocytes in patients with renal cell or bladder cancers. These studies demonstrated the therapeutic potential of optimizing T cell-based immunotherapy for STEAP-expressing renal cells and bladder cancer (Azumi et al., 2010).

Producing an effective vaccine is one of the most important goals of tumor immunotherapy. It has been found that mouse STEAP (mSTEAP)-based vaccination can induce a specific CD8 T cell response to newly defined mSTEAP epitopes and prolong the survival rate of tumor-challenged mice, showing that vaccination against mSTEAP is a feasible option to delay tumor growth (Garcia-Hernandez Mde et al., 2007). Similarly, Cappuccini et al. (2016) constructed a simian adenovirus and modified a vaccinia Ankara virus, ChAdOx1-MVA, encoding STEAP1, to induce strong, sustained antigen-specific CD8+ T-cell responses in male C57BL/6 and BALB/c mice, which when combined with PD-1 blocking antibody, significantly improved the survival rate of animals, indicating that a ChAdOx1-MVA vaccination regimen for STEAP1 combined with PD-1 treatment may have high therapeutic potential in the clinic. Machlenkin et al. (2005) identified two novel immunogenic peptides derived from overexpressed prostate antigens, prostatic acid phosphatase (PAP) and STEAP1, and found that they could induce peptide-specific CTLs targeting human leukocyte antigen-A2.1^+^ LNCaP cells to inhibit tumor growth in adoptive immunotherapy, providing great potential as candidate vaccines for patients with prostate cancers.

A mAb targeting STEAP4 has been shown to promote caspase-dependent apoptosis and inhibit the proliferation of pre-adipocytes and glucose uptake without affecting lipogenesis and lipogenic differentiation (Qin et al., 2010). Since mAb binding to STEAPs appears to inhibit the function of STEAPs, it is easy to speculate that specific small molecules that may interfere with STEAP ion channels or their active sites may impair their carcinogenic function and may therefore also serve as targeted therapeutic agents.

Although the current therapeutic strategies for STEAPs have not been used in the clinic, their role in molecular transport and involvement in cancer progression makes them promising targets in the treatment of patients with prostate cancer, and even other types of cancers. Overall, future immunotherapeutic strategies are likely to include all members of the STEAP family.

## Conclusion

Six-transmembrane epithelial antigen of the prostate family members are very similar in structure and, except for STEAP1, all serve as metal oxidoreductases in the absorption of copper and iron. STEAP family members in different cancers are irregularly expressed and participate in the proliferation, migration, invasion, apoptosis, and prognosis of cancer cells, although STEAP3 and STEAP4 may have tumor suppressor functions in cancers. All in all, current studies clearly indicate that the members of the STEAP family are likely to become biomarkers of cancer diseases. Although the mechanisms of the STEAPs in the progression of different cancers are still largely elusive, it is clear that the STEAP family is closely related to the occurrence and development of tumors. Therefore, further investigations are needed to clarify the role of STEAPs in tumor cell proliferation, cell cycling, apoptosis, invasion, and metastasis. In addition, more attention should be paid to the regulatory mechanisms of STEAP family members in different cancers. Future work must systematically analyze and describe the STEAP family based on molecular, cellular, animal and clinical data, and further explore their regulation and role in cancer pathophysiology, diagnosis, and treatment.

## Author Contributions

JL and W-JC: conceptualization. W-JC, H-TW, C-LL, Y-KL, Z-XF, and W-TL: organization of the database. W-JC, H-TW, C-LL, and Y-KL: searching the literature. W-JC and H-TW: writing – original draft preparation. JL, C-LL, Y-KL, Z-XF, and W-TL: writing – review and editing. JL: supervision and project administration. JL and H-TW: funding acquisition. All authors have read and agreed to the published version of the manuscript.

## Conflict of Interest

The authors declare that the research was conducted in the absence of any commercial or financial relationships that could be construed as a potential conflict of interest.

## Publisher’s Note

All claims expressed in this article are solely those of the authors and do not necessarily represent those of their affiliated organizations, or those of the publisher, the editors and the reviewers. Any product that may be evaluated in this article, or claim that may be made by its manufacturer, is not guaranteed or endorsed by the publisher.

## References

[B1] Azumi M. Kobayashi H. Aoki N. Sato K. Kimura S. Kakizaki H. (2010). Six-transmembrane epithelial antigen of the prostate as an immunotherapeutic target for renal cell and bladder cancer. *J. Urol.* 183 2036–2044. 10.1016/j.juro.2009.12.094 20303532

[B2] Barresi V. Trovato-Salinaro A. Spampinato G. Musso N. Castorina S. Rizzarelli E. (2016). Transcriptome analysis of copper homeostasis genes reveals coordinated upregulation of SLC31A1,SCO1, and COX11 in colorectal cancer. *FEBS Open Bio* 6 794–806. 10.1002/2211-5463.12060 27516958 PMC4971835

[B3] Barroca-Ferreira J. Pais J. P. Santos M. M. Goncalves A. M. Gomes I. M. Sousa I. (2018). Targeting STEAP1 protein in human cancer: current trends and future challenges. *Curr. Cancer Drug Targets* 18 222–230. 10.2174/1568009617666170427103732 28460619

[B4] Bhatlekar S. Addya S. Salunek M. Orr C. R. Surrey S. Mckenzie S. (2014). Identification of a developmental gene expression signature, including HOX genes, for the normal human colonic crypt stem cell niche: overexpression of the signature parallels stem cell overpopulation during colon tumorigenesis. *Stem Cells Dev.* 23 167–179. 10.1089/scd.2013.0039 23980595 PMC3887463

[B5] Boelens M. C. Van Den Berg A. Fehrmann R. S. Geerlings M. De Jong W. K. Te Meerman G. J. (2009). Current smoking-specific gene expression signature in normal bronchial epithelium is enhanced in squamous cell lung cancer. *J. Pathol.* 218 182–191. 10.1002/path.2520 19334046

[B6] Boswell C. A. Mundo E. E. Zhang C. Bumbaca D. Valle N. R. Kozak K. R. (2011). Impact of drug conjugation on pharmacokinetics and tissue distribution of anti-STEAP1 antibody-drug conjugates in rats. *Bioconjug. Chem.* 22 1994–2004. 10.1021/bc200212a 21913715

[B7] Burnell S. E. A. Spencer-Harty S. Howarth S. Bodger O. Kynaston H. Morgan C. (2018). STEAP2 knockdown reduces the invasive potential of prostate cancer cells. *Sci. Rep.* 8:6252. 10.1038/s41598-018-24655-x 29674723 PMC5908900

[B8] Burnell S. E. A. Spencer-Harty S. Howarth S. Bodger O. Kynaston H. Morgan C. (2019). Utilisation of the STEAP protein family in a diagnostic setting may provide a more comprehensive prognosis of prostate cancer. *PLoS One* 14:e0220456. 10.1371/journal.pone.0220456 31393902 PMC6687176

[B9] Cadiou J. L. Pichat S. Bondanese V. P. Soulard A. Fujii T. Albarede F. (2017). Copper transporters are responsible for copper isotopic fractionation in eukaryotic cells. *Sci. Rep.* 7:44533. 10.1038/srep44533 28303916 PMC5356015

[B10] Caillot F. Daveau R. Daveau M. Lubrano J. Saint-Auret G. Hiron M. (2009). Down-regulated expression of the TSAP6 protein in liver is associated with a transition from cirrhosis to hepatocellular carcinoma. *Histopathology* 54 319–327. 10.1111/j.1365-2559.2009.03224.x 19236508

[B11] Cappuccini F. Stribbling S. Pollock E. Hill A. V. Redchenko I. (2016). Immunogenicity and efficacy of the novel cancer vaccine based on simian adenovirus and MVA vectors alone and in combination with PD-1 mAb in a mouse model of prostate cancer. *Cancer Immunol. Immunother.* 65 701–713. 10.1007/s00262-016-1831-8 27052571 PMC4880633

[B12] Challita-Eid P. M. Morrison K. Etessami S. An Z. Morrison K. J. Perez-Villar J. J. (2007). Monoclonal antibodies to six-transmembrane epithelial antigen of the prostate-1 inhibit intercellular communication in vitro and growth of human tumor xenografts in vivo. *Cancer Res.* 67 5798–5805. 10.1158/0008-5472.CAN-06-3849 17575147

[B13] Esmaeili S. A. Nejatollahi F. Sahebkar A. (2018). Inhibition of intercellular communication between prostate cancer cells by a specific anti-STEAP-1 single chain antibody. *Anticancer Agents Med. Chem.* 18 1674–1679. 10.2174/1871520618666171208092115 29219059

[B14] Finegold A. A. Shatwell K. P. Segal A. W. Klausner R. D. Dancis A. (1996). Intramembrane bis-heme motif for transmembrane electron transport conserved in a yeast iron reductase and the human NADPH oxidase. *J. Biol. Chem.* 271 31021–31024. 10.1074/jbc.271.49.31021 8940093

[B15] Garcia-Hernandez Mde L. Gray A. Hubby B. Kast W. M. (2007). In vivo effects of vaccination with six-transmembrane epithelial antigen of the prostate: a candidate antigen for treating prostate cancer. *Cancer Res.* 67 1344–1351. 10.1158/0008-5472.CAN-06-2996 17283172

[B16] Gelfand R. Vernet D. Bruhn K. W. Sarkissyan S. Heber D. Vadgama J. V. (2017). Long-term exposure of MCF-7 breast cancer cells to ethanol stimulates oncogenic features. *Int. J. Oncol.* 50 49–65. 10.3892/ijo.2016.3800 27959387 PMC5182011

[B17] Gomes I. M. Maia C. J. Santos C. R. (2012). STEAP proteins: from structure to applications in cancer therapy. *Mol. Cancer Res.* 10 573–587. 10.1158/1541-7786.MCR-11-0281 22522456

[B18] Gomes I. M. Rocha S. M. Gaspar C. Alvelos M. I. Santos C. R. Socorro S. (2018). Knockdown of STEAP1 inhibits cell growth and induces apoptosis in LNCaP prostate cancer cells counteracting the effect of androgens. *Med. Oncol.* 35:40. 10.1007/s12032-018-1100-0 29464393

[B19] Gomes I. M. Santos C. R. Maia C. J. (2014b). Expression of STEAP1 and STEAP1B in prostate cell lines, and the putative regulation of STEAP1 by post-transcriptional and post-translational mechanisms. *Genes Cancer* 5 142–151. 10.18632/genesandcancer.13 25053991 PMC4091532

[B20] Gomes I. M. Arinto P. Lopes C. Santos C. R. Maia C. J. (2014a). STEAP1 is overexpressed in prostate cancer and prostatic intraepithelial neoplasia lesions, and it is positively associated with Gleason score. *Urol. Oncol.* 32 e23–e59. 10.1016/j.urolonc.2013.08.028 24239460

[B21] Gomes I. M. Santos C. R. Socorro S. Maia C. J. (2013). Six transmembrane epithelial antigen of the prostate 1 is down-regulated by sex hormones in prostate cells. *Prostate* 73 605–613. 10.1002/pros.22601 23060075

[B22] Gonen-Korkmaz C. Sevin G. Gokce G. Arun M. Z. Yildirim G. Reel B. (2014). Analysis of tumor necrosis factor alpha-induced and nuclear factor kappaB-silenced LNCaP prostate cancer cells by RT-qPCR. *Exp. Ther. Med.* 8 1695–1700. 10.3892/etm.2014.2032 25371717 PMC4218634

[B23] Grunewald T. G. Bach H. Cossarizza A. Matsumoto I. (2012a). The STEAP protein family: versatile oxidoreductases and targets for cancer immunotherapy with overlapping and distinct cellular functions. *Biol. Cell* 104 641–657. 10.1111/boc.201200027 22804687

[B24] Grunewald T. G. Diebold I. Esposito I. Plehm S. Hauer K. Thiel U. (2012b). STEAP1 is associated with the invasive and oxidative stress phenotype of Ewing tumors. *Mol. Cancer Res.* 10 52–65. 10.1158/1541-7786.MCR-11-0524 22080479

[B25] Grunewald T. G. Ranft A. Esposito I. Da Silva-Buttkus P. Aichler M. Baumhoer D. (2012c). High STEAP1 expression is associated with improved outcome of Ewing’s sarcoma patients. *Ann. Oncol.* 23 2185–2190. 10.1093/annonc/mdr605 22317770

[B26] Guo H. He J. Yang X. Zheng W. Yao W. (2020). Responses of intestinal morphology and function in offspring to heat stress in primiparous sows during late gestation. *J. Therm. Biol.* 89:102539. 10.1016/j.jtherbio.2020.102539 32364966

[B27] Guo Q. Ke X. X. Liu Z. Gao W. L. Fang S. X. Chen C. (2020). Evaluation of the prognostic value of STEAP1 in lung adenocarcinoma and insights into its potential molecular pathways via bioinformatic analysis. *Front. Genet.* 11:242. 10.3389/fgene.2020.00242 32265985 PMC7099762

[B28] Han M. Xu R. Wang S. Yang N. Ni S. Zhang Q. (2018). Six-transmembrane epithelial antigen of prostate 3 predicts poor prognosis and promotes glioblastoma growth and invasion. *Neoplasia* 20 543–554. 10.1016/j.neo.2018.04.002 29730475 PMC5994776

[B29] Hayashi T. Oue N. Sakamoto N. Anami K. Oo H. Z. Sentani K. (2011). Identification of transmembrane protein in prostate cancer by the *Escherichia coli* ampicillin secretion trap: expression of CDON is involved in tumor cell growth and invasion. *Pathobiology* 78 277–284. 10.1159/000329588 21849809

[B30] Hubert R. S. Vivanco I. Chen E. Rastegar S. Leong K. Mitchell S. C. (1999). STEAP: a prostate-specific cell-surface antigen highly expressed in human prostate tumors. *Proc. Natl. Acad. Sci. U. S. A.* 96 14523–14528. 10.1073/pnas.96.25.14523 10588738 PMC24469

[B31] Ihlaseh-Catalano S. M. Drigo S. A. De Jesus C. M. Domingues M. A. Trindade Filho J. C. De Camargo J. L. (2013). STEAP1 protein overexpression is an independent marker for biochemical recurrence in prostate carcinoma. *Histopathology* 63 678–685. 10.1111/his.12226 24025158

[B32] Iijima K. Nakamura H. Takada K. Hayasaka N. Kubo T. Umeyama Y. (2021). Six-transmembrane epithelial antigen of the prostate 1 accelerates cell proliferation by targeting c-Myc in liver cancer cells. *Oncol. Lett.* 22:546. 10.3892/ol.2021.12807 34335918 PMC8316717

[B33] Isobe T. Baba E. Arita S. Komoda M. Tamura S. Shirakawa T. (2011). Human STEAP3 maintains tumor growth under hypoferric condition. *Exp. Cell Res.* 317 2582–2591. 10.1016/j.yexcr.2011.07.022 21871451

[B34] Jiang C. Wu B. Xue M. Lin J. Hu Z. Nie X. (2021). Inflammation accelerates copper-mediated cytotoxicity through induction of six-transmembrane epithelial antigens of prostate 4 expression. *Immunol. Cell Biol.* 99 392–402. 10.1111/imcb.12427 33179273

[B35] Jiang J. N. Wu Y. Y. Fang X. D. Ji F. J. (2020). EIF4E regulates STEAP1 expression in peritoneal metastasis. *J. Cancer* 11 990–996. 10.7150/jca.29105 31949502 PMC6959031

[B36] Jiao Z. Huang L. Sun J. Xie J. Wang T. Yin X. (2020). Six-transmembrane epithelial antigen of the prostate 1 expression promotes ovarian cancer metastasis by aiding progression of epithelial-to-mesenchymal transition. *Histochem. Cell Biol.* 154 215–230. 10.1007/s00418-020-01877-7 32382787

[B37] Jin Y. Wang L. Qu S. Sheng X. Kristian A. Maelandsmo G. M. (2015). STAMP2 increases oxidative stress and is critical for prostate cancer. *EMBO Mol. Med.* 7 315–331. 10.15252/emmm.201404181 25680860 PMC4364948

[B38] Kim S. H. Ho J. N. Jin H. Lee S. C. Lee S. E. Hong S. K. (2016). Upregulated expression of BCL2, MCM7, and CCNE1 indicate cisplatin-resistance in the set of two human bladder cancer cell lines: T24 cisplatin sensitive and T24R2 cisplatin resistant bladder cancer cell lines. *Investig. Clin. Urol.* 57 63–72. 10.4111/icu.2016.57.1.63 26966728 PMC4778756

[B39] Knutson M. D. (2007). Steap proteins: implications for iron and copper metabolism. *Nutr. Rev.* 65 335–340. 17695374 10.1111/j.1753-4887.2007.tb00311.x

[B40] Korkmaz C. G. Korkmaz K. S. Kurys P. Elbi C. Wang L. Klokk T. I. (2005). Molecular cloning and characterization of STAMP2, an androgen-regulated six transmembrane protein that is overexpressed in prostate cancer. *Oncogene* 24 4934–4945. 10.1038/sj.onc.1208677 15897894

[B41] Korkmaz K. S. Elbi C. Korkmaz C. G. Loda M. Hager G. L. Saatcioglu F. (2002). Molecular cloning and characterization of STAMP1, a highly prostate-specific six transmembrane protein that is overexpressed in prostate cancer. *J. Biol. Chem.* 277 36689–36696. 10.1074/jbc.M202414200 12095985

[B42] Lan G. Yu X. Sun X. Li W. Zhao Y. Lan J. (2021). Comprehensive analysis of the expression and prognosis for TNFAIPs in head and neck cancer. *Sci. Rep.* 11:15696. 10.1038/s41598-021-95160-x 34344926 PMC8333337

[B43] Lee C. H. Chen S. L. Sung W. W. Lai H. W. Hsieh M. J. Yen H. H. (2016). The prognostic role of STEAP1 expression determined via immunohistochemistry staining in predicting prognosis of primary colorectal cancer: a survival analysis. *Int. J. Mol. Sci.* 17:592. 10.3390/ijms17040592 27104516 PMC4849046

[B44] Lespagnol A. Duflaut D. Beekman C. Blanc L. Fiucci G. Marine J. C. (2008). Exosome secretion, including the DNA damage-induced p53-dependent secretory pathway, is severely compromised in TSAP6/Steap3-null mice. *Cell Death Differ.* 15 1723–1733. 10.1038/cdd.2008.104 18617898

[B45] Li L. Li J. Shen Z. Liu W. Chen Z. (2004). [Clinical significance of six-transmembrane epithelial antigen of the prostate expressed in prostatic carcinoma]. *Zhonghua Nan Ke Xue* 10 351–354. 15190827

[B46] Liao Y. Zhao J. Bulek K. Tang F. Chen X. Cai G. (2020). Inflammation mobilizes copper metabolism to promote colon tumorigenesis via an IL-17-STEAP4-XIAP axis. *Nat. Commun.* 11:900. 10.1038/s41467-020-14698-y 32060280 PMC7021685

[B47] Liu Z. Zhang H. Hu H. Cai Z. Lu C. Liang Q. (2021). A novel six-mRNA signature predicts survival of patients with glioblastoma multiforme. *Front. Genet.* 12:634116. 10.3389/fgene.2021.634116 33790946 PMC8006298

[B48] Machlenkin A. Paz A. Bar Haim E. Goldberger O. Finkel E. Tirosh B. (2005). Human CTL epitopes prostatic acid phosphatase-3 and six-transmembrane epithelial antigen of prostate-3 as candidates for prostate cancer immunotherapy. *Cancer Res.* 65 6435–6442. 10.1158/0008-5472.CAN-05-0133 16024648

[B49] Maia C. J. Socorro S. Schmitt F. Santos C. R. (2008). STEAP1 is over-expressed in breast cancer and down-regulated by 17beta-estradiol in MCF-7 cells and in the rat mammary gland. *Endocrine* 34 108–116. 10.1007/s12020-008-9113-7 18958632

[B50] Moreaux J. Kassambara A. Hose D. Klein B. (2012). STEAP1 is overexpressed in cancers: a promising therapeutic target. *Biochem. Biophys. Res. Commun.* 429 148–155. 10.1016/j.bbrc.2012.10.123 23142226

[B51] Nakamura H. Takada K. Arihara Y. Hayasaka N. Murase K. Iyama S. (2019). Six-transmembrane epithelial antigen of the prostate 1 protects against increased oxidative stress via a nuclear erythroid 2-related factor pathway in colorectal cancer. *Cancer Gene Ther.* 26 313–322. 10.1038/s41417-018-0056-8 30401882

[B52] Ohgami R. S. Campagna D. R. Greer E. L. Antiochos B. Mcdonald A. Chen J. (2005). Identification of a ferrireductase required for efficient transferrin-dependent iron uptake in erythroid cells. *Nat. Genet.* 37 1264–1269. 10.1038/ng1658 16227996 PMC2156108

[B53] Ohgami R. S. Campagna D. R. Mcdonald A. Fleming M. D. (2006). The steap proteins are metalloreductases. *Blood* 108 1388–1394. 10.1182/blood-2006-02-003681 16609065 PMC1785011

[B54] Orfanou I. M. Argyros O. Papapetropoulos A. Tseleni-Balafouta S. Vougas K. Tamvakopoulos C. (2021). Discovery and pharmacological evaluation of STEAP4 as a novel target for HER2 overexpressing breast cancer. *Front. Oncol.* 11:608201. 10.3389/fonc.2021.608201 33842315 PMC8034292

[B55] Passer B. J. Nancy-Portebois V. Amzallag N. Prieur S. Cans C. Roborel De Climens A. (2003). The p53-inducible TSAP6 gene product regulates apoptosis and the cell cycle and interacts with Nix and the Myt1 kinase. *Proc. Natl. Acad. Sci. U. S. A.* 100 2284–2289. 10.1073/pnas.0530298100 12606722 PMC151332

[B56] Pihlstrom N. Jin Y. Nenseth Z. Kuzu O. F. Saatcioglu F. (2021). STAMP2 expression mediated by cytokines attenuates their growth-limiting effects in prostate cancer cells. *Cancers* 13:1579. 10.3390/cancers13071579 33808059 PMC8036285

[B57] Porkka K. P. Helenius M. A. Visakorpi T. (2002). Cloning and characterization of a novel six-transmembrane protein STEAP2, expressed in normal and malignant prostate. *Lab. Invest.* 82 1573–1582. 10.1097/01.LAB.0000038554.26102.C6 12429817

[B58] Porkka K. P. Nupponen N. N. Tammela T. L. Vessella R. L. Visakorpi T. (2003). Human pHyde is not a classical tumor suppressor gene in prostate cancer. *Int. J. Cancer* 106 729–735. 10.1002/ijc.11278 12866033

[B59] Qin D. N. Kou C. Z. Ni Y. H. Zhang C. M. Zhu J. G. Zhu C. (2010). Monoclonal antibody to the six-transmembrane epithelial antigen of prostate 4 promotes apoptosis and inhibits proliferation and glucose uptake in human adipocytes. *Int. J. Mol. Med.* 26 803–811. 10.3892/ijmm_00000528 21042773

[B60] Rinaldy A. R. Steiner M. S. (1999). Application of an improved cDNA competition technique to identify prostate cancer-associated gene. *DNA Cell Biol.* 18 829–836. 10.1089/104454999314827 10595396

[B61] Savci-Heijink C. D. Halfwerk H. Koster J. Van De Vijver M. J. (2016). A novel gene expression signature for bone metastasis in breast carcinomas. *Breast Cancer Res. Treat.* 156 249–259. 10.1007/s10549-016-3741-z 26965286 PMC4819548

[B62] Sun J. Ji G. Xie J. Jiao Z. Zhang H. Chen J. (2019). Six-transmembrane epithelial antigen of the prostate 1 is associated with tumor invasion and migration in endometrial carcinomas. *J. Cell Biochem.* 120 11172–11189. 10.1002/jcb.28393 30714206

[B63] Tian Y. X. Huo S. F. Shang W. L. Yu M. Ren X. P. Wen H. X. (2020). STEAP1 facilitates metastasis and epithelial-mesenchymal transition of lung adenocarcinoma via the JAK2/STAT3 signaling pathway. *Biosci. Rep.* 40:BSR20193169. 10.1042/BSR20193169 32515474 PMC7300283

[B64] Valadi H. Ekstrom K. Bossios A. Sjostrand M. Lee J. J. Lotvall J. O. (2007). Exosome-mediated transfer of mRNAs and microRNAs is a novel mechanism of genetic exchange between cells. *Nat. Cell Biol.* 9 654–659. 10.1038/ncb1596 17486113

[B65] von Rozycki T. Yen M. R. Lende E. E. Saier M. H. Jr. (2004). The YedZ family: possible heme binding proteins that can be fused to transporters and electron carriers. *J. Mol. Microbiol. Biotechnol.* 8 129–140. 10.1159/000085786 16088215

[B66] Wang L. Jin Y. Arnoldussen Y. J. Jonson I. Qu S. Maelandsmo G. M. (2010). STAMP1 is both a proliferative and an antiapoptotic factor in prostate cancer. *Cancer Res.* 70 5818–5828. 10.1158/0008-5472.CAN-09-4697 20587517

[B67] Weston C. Klobusicky J. Weston J. Connor J. Toms S. A. Marko N. F. (2016). Aberrations in the iron regulatory gene signature are associated with decreased survival in diffuse infiltrating gliomas. *PLoS One* 11:e0166593. 10.1371/journal.pone.0166593 27898674 PMC5127508

[B68] Whiteland H. Spencer-Harty S. Morgan C. Kynaston H. Thomas D. H. Bose P. (2014). A role for STEAP2 in prostate cancer progression. *Clin. Exp. Metastasis* 31 909–920. 10.1007/s10585-014-9679-9 25248617

[B69] Wong P. F. Abubakar S. (2010). Comparative transcriptional study of the effects of high intracellular zinc on prostate carcinoma cells. *Oncol. Rep.* 23 1501–1516. 10.3892/or_00000789 20428803

[B70] Wu H. T. Chen W. J. Xu Y. Shen J. X. Chen W. T. Liu J. (2020). The tumor suppressive roles and prognostic values of STEAP family members in breast cancer. *Biomed. Res. Int.* 2020:9578484. 10.1155/2020/9578484 32802887 PMC7421016

[B71] Wu Y. Y. Jiang J. N. Fang X. D. Ji F. J. (2018). STEAP1 regulates tumorigenesis and chemoresistance during peritoneal metastasis of gastric cancer. *Front. Physiol.* 9:1132. 10.3389/fphys.2018.01132 30246786 PMC6110897

[B72] Xie J. Yang Y. Sun J. Jiao Z. Zhang H. Chen J. (2019). STEAP1 inhibits breast cancer metastasis and is associated with epithelial-mesenchymal transition procession. *Clin. Breast Cancer* 19 e195–e207. 10.1016/j.clbc.2018.08.010 30253922

[B73] Xue X. Bredell B. X. Anderson E. R. Martin A. Mays C. Nagao-Kitamoto H. (2017). Quantitative proteomics identifies STEAP4 as a critical regulator of mitochondrial dysfunction linking inflammation and colon cancer. *Proc. Natl. Acad. Sci. U. S. A.* 114 E9608–E9617. 10.1073/pnas.1712946114 29078383 PMC5692584

[B74] Yamamoto T. Tamura Y. Kobayashi J. Kamiguchi K. Hirohashi Y. Miyazaki A. (2013). Six-transmembrane epithelial antigen of the prostate-1 plays a role for in vivo tumor growth via intercellular communication. *Exp. Cell Res.* 319 2617–2626. 10.1016/j.yexcr.2013.07.025 23916873

[B75] Yan D. Dong W. He Q. Yang M. Huang L. Kong J. (2019). Circular RNA circPICALM sponges miR-1265 to inhibit bladder cancer metastasis and influence FAK phosphorylation. *EBioMedicine* 48 316–331. 10.1016/j.ebiom.2019.08.074 31648990 PMC6838432

[B76] Yang Q. Ji G. Li J. (2020). STEAP2 is down-regulated in breast cancer tissue and suppresses PI3K/AKT signaling and breast cancer cell invasion in vitro and in vivo. *Cancer Biol. Ther.* 21 278–291. 10.1080/15384047.2019.1685290 31696760 PMC7012168

[B77] Yu Z. Wang H. Fang Y. Lu L. Li M. Yan B. (2020). Molecular chaperone HspB2 inhibited pancreatic cancer cell proliferation via activating p53 downstream gene RPRM. BAI1, and TSAP6. *J. Cell Biochem.* 121 2318–2329. 10.1002/jcb.29455 31692031

[B78] Yuan Y. Liu Y. Zhu X. M. Hu J. Zhao C. Y. Jiang F. (2019). Six-transmembrane epithelial antigen of the prostate-1 (STEAP-1)-targeted ultrasound imaging microbubble improves detection of prostate cancer in vivo. *J. Ultrasound Med.* 38 299–305. 10.1002/jum.14689 30027616

[B79] Zhang M. Lv X. Jiang Y. Li G. Qiao Q. (2019). Identification of aberrantly methylated differentially expressed genes in glioblastoma multiforme and their association with patient survival. *Exp. Ther. Med.* 18 2140–2152. 10.3892/etm.2019.7807 31452706 PMC6704589

[B80] Zhang X. Steiner M. S. Rinaldy A. Lu Y. (2001). Apoptosis induction in prostate cancer cells by a novel gene product, pHyde, involves caspase-3. *Oncogene* 20 5982–5990. 10.1038/sj.onc.1204831 11593405

[B81] Zhang Y. Ye Q. He J. Chen P. Wan J. Li J. (2020). Recurrence-associated multi-RNA signature to predict disease-free survival for ovarian cancer patients. *Biomed. Res. Int.* 2020:1618527. 10.1155/2020/1618527 32149080 PMC7044477

[B82] Zhuang X. Herbert J. M. Lodhia P. Bradford J. Turner A. M. Newby P. M. (2015). Identification of novel vascular targets in lung cancer. *Br. J. Cancer* 112 485–494. 10.1038/bjc.2014.626 25535734 PMC4453649

